# Bilateral anterior ischemic optic neuropathy in patients on dialysis: A report of two cases

**DOI:** 10.4103/0971-4065.62094

**Published:** 2010

**Authors:** J. Nieto, M.A. Zapata

**Affiliations:** 1 Consultores en cirugía Oculoplástica, Hospital Vall d'Hebrón, Barcelona, Spain; 2 Ophthalmology Service, Hospital de Sant Joan Despí, Consorci Sanitari Integral, Hospital Vall d'Hebrón, Barcelona, Spain; 3 Ophthalmology Service, Hospital Vall d'Hebrón, Barcelona, Spain

**Keywords:** Anterior ischemic optic neuropathy, bilateral anterior ischemic optic neuropathy, visual loss in dialysis, visual loss in uremia

## Abstract

Patients under chronic dialysis treatment suffer from atherosclerotic disease and anemia more frequently than the normal population. This, together with the frequent hypotension, put these patients at increased risk for anterior ischemic optic neuropathy (AION), which may be bilateral and blinding. We present two cases of patients under chronic dialysis who developed bilateral AION after hypotensive events. Bilateral involvement is not unusual in renal replacement patients who suffer from AION. Efforts should be focused on prevention of this complication by improving anemia and blood pressure control because once established, treatment will probably be ineffective.

## Introduction

Non-arteritic anterior ischemic optic neuropathy (AION) is the most common acute optic neuropathy in patients over 50 years of age.[1] Fellow eye involvement has been reported in 15% of patients at five years, according to one study.[2] However, bilateral simultaneous AION is rare and frequently associated to severe hypotensive situations.[3,4]

Patients under renal replacement therapy may be at special risk for developing AION.[5–12] It is well known how patients under long-term dialysis are prone to accelerated atherosclerotic disease,[13] which, together with anemia, are known predisposing factors for this condition. Hypotension, on the other hand, seems to be the most common precipitating factor, both in the normal and the dialysis population.[5–10]

We present two cases of bilateral, blinding AION that show how hypotension may precipitate this devastating complication in both hemodialysis and peritoneal dialysis patients.

## Case Reports

### Case 1

**Figure 1:**
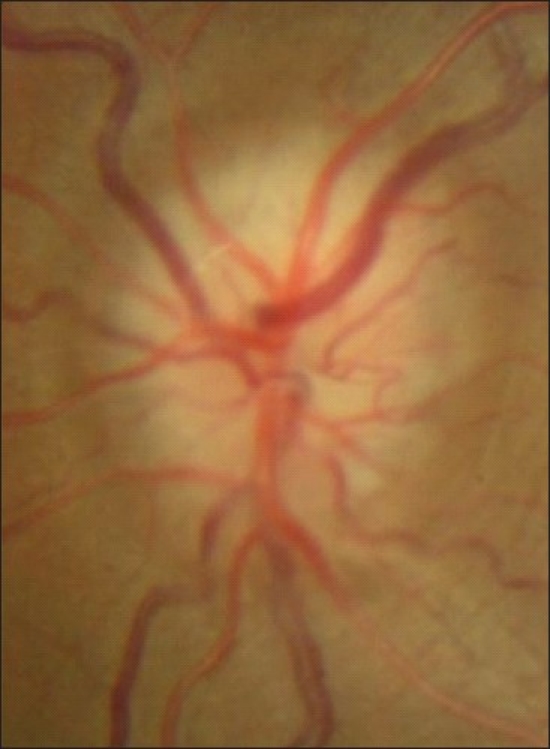
Left optic nerve at presentation, showing diffuse swelling

**Figure 2:**
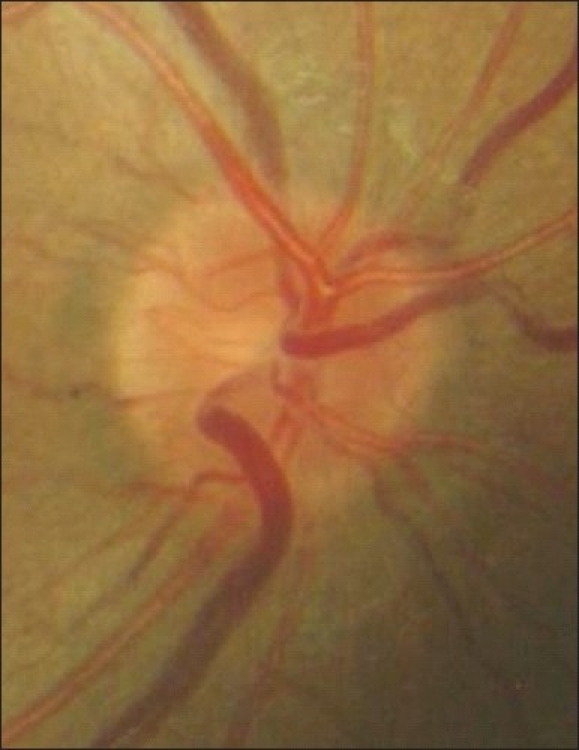
Normal right optic nerve at presentation

**Figure 3:**
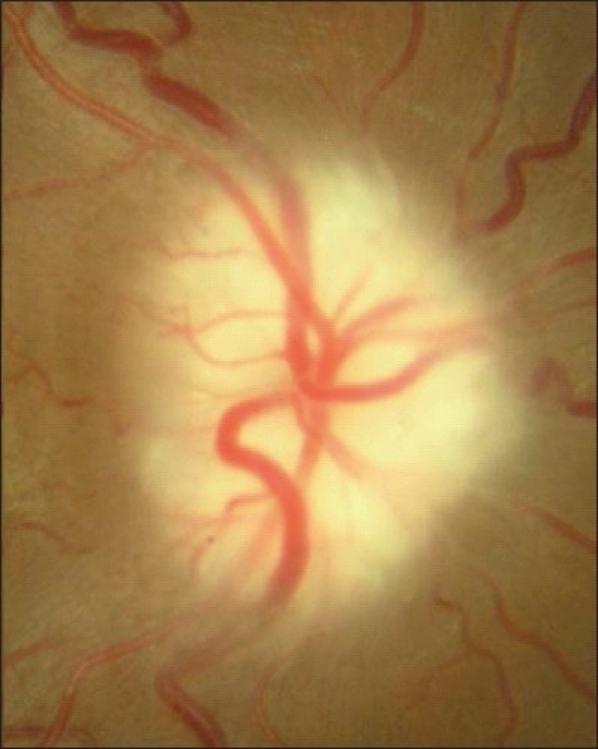
Two weeks later, right optic nerve showing very pale swelling

A 26 year-old male, under hemodialysis since the age of six for end stage renal disease secondary to urethral diverticle, with grade IV vesico urethral reflux with hidronephrosis. Renal transplant had failed at the age of seven. He had long-standing arterial hypotension, with usual blood pressures of 70/50 mmHg, and chronic anemia for which he was being treated with erythropoietin. His serum creatinine was 9 mg/dl between dialysis sessions. He presented to the ER complaining of painless visual loss in his left eye after hemodialysis. On that same day he had suffered a hypotensive event during his dialysis session, with transient binocular visual loss. His visual acuity was: R.E 20/30, L.E 20/200, with left RAPD. His left optic nerve was pale and swollen [Figure 1], being the right optic nerve normal [Figure 2]. A diagnosis of AION was made. He received no treatment, and on the following days his left eye evolved to no light perception. Poor collaboration prevented us from visual field testing. His nephrologist was contacted and, again, special care was given to blood pressure control. Etilefrin was also given to achieve higher blood pressures during sessions. However, one week later he presented with reduced visual acuity in his right eye after hypotension during hemodialysis. His right optic nerve was then pale and swollen [Figure 3], and evolved to no light perception within a few days in spite of oral steroids. Tests for Leber's optic neuropathy returned negative.

### Case 2

A 56-year-old male under nocturnal ambulatory peritoneal dialysis for end stage renal disease of unknown cause presented to the ER complaining of bilateral painless visual loss on awakening. His visual acuity was: Right eye (R.E):20/400, left eye (L.E): Hand movement. His pupils were poorly reactive, without RAPD. Fundoscopy revealed bilateral pale swelling of the optic nerves, with two splinter hemorrhages on the left. Visual field testing showed a typical inferior altitudinal defect on his right eye and was not possible to obtain in the left. Arteritic and uremic neuropathies were ruled out on the basis of the clinical picture and his analytic parameters. His serum creatinine was 8 mg/dl. He referred repeated morning arterial blood pressures of 80-90/50-60 mmHg. The patient was urgently referred to his nephrologist, who switched him to hemodialysis and achieved a better blood pressure control. Oral steroids (60 mg prednisone daily) were also prescribed and tapered over six weeks. In spite of these measures, vision declined to counting fingers in his right eye and remained stable in the left.

## Discussion

Acute visual loss caused by optic neuropathy in patients under chronic dialysis challenges the ophthalmologist to diagnose mainly between ischemic and uremic optic neuropathies. Non-arteritic anterior ischemic optic neuropathy (AION) typically presents as sudden, painless visual loss that many patients report on awakening, accompanied by an afferent pupillary defect and optic disc edema. Visual field testing most frequently shows nerve fiber bundle defects, being inferior altitudinal the most usual.[1] Bilateral simultaneous AION is extremely rare in normal patients if not related to severe blood loss during long surgeries or trauma. Bilateral involvement is however very common in chronic dialysis patients who suffer from AION; of the 12 cases reported (together with ours) in adults and not related to optic disc drusen, nine were bilateral.[5–12]

Uremic optic neuropathy, on the other hand, is an infrequent condition that affects patients with extremely high renal parameters, which is rare in chronic dialysis patients. It is usually found in subjects with undiagnosed renal disease. It has been postulated that direct toxicity to the optic nerve by dialyzable metabolites would cause a visual impairment that would be reversible if cleared soon enough from the patient's serum. Knox reported significant improvement in five of six patients who underwent prompt treatment with hemodialysis and steroids.[14] Both our patients had serum creatinine within reasonable limits, which together with the clinical picture made us think of AION.

Toxic optic neuropathy is another concern in these patients. Although only anecdotally, it has been reported with the use of desferrioxamine and OKT3 in dialysis patients.[15–17] This is a very rare entity and should always be an exclusion diagnosis. It is our opinion that AION should always be the first suspicion in a dialyzed patient with an acute optic neuropathy.

It is well known that patients under dialysis are prone to hypotensive events. Reviewing the scarce literature on AION and dialysis, we find that most cases have been related to hypotension.[5–12] Peritoneal dialysis, because of its continuous nature, would theoretically have less risk of blood pressure drops, but the cases reported are distributed equally into both the peritoneal and hemodialysis groups. Hypotension was clearly the precipitating factor in both our patients. Although we did not obtain ambulatory monitoring in our first patient, he reported visual loss on awakening, and had repeatedly low morning arterial blood pressure recordings. Hayreh described nocturnal hypotension as the most important precipitating factor in the pathogenesis of AION in normal patients, and it is nowadays the best explanation to why so many patients with AION complain of visual loss an awakening.[18] Interestingly, Jackson described ambulatory blood pressure monitoring in a patient under peritoneal dialysis who suffered bilateral AION, showing severe drops in nocturnal blood pressure, with diastolic readings of as low as 41 mmHg.[9]

Although Conolly reported moderate visual recovery in hypotensive AION following vigorous blood pressure raise,[6] most published reports on hypotensive AION (dialysis related or not) have found treatment far from satisfactory.[7–12] Systemic steroids are often prescribed in AION but there is no solid evidence that favor their use. When dealing with patients under chronic dialysis, prevention of severe hypotensive events is of outmost importance, and nephrologists should be aware of this potentially blinding disease. Even though we were unfortunately unable to protect the right eye of our second patient, achieving the best possible control of both anemia and blood pressure is probably the only way of preventing this catastrophic disorder. Similar to AION in giant cell arteritis patients, we believe acute unilateral AION in a renal replacement patient should be considered an interdisciplinary emergency with high risk of developing AION in the fellow eye.
